# Performance Comparison of Three Fibre-Based Reflective Optical Sensors for Aero Engine Monitorization

**DOI:** 10.3390/s19102244

**Published:** 2019-05-15

**Authors:** Rubén Fernández-Bello, Josu Amorebieta, Josu Beloki, Gotzon Aldabaldetreku, Iker García, Joseba Zubia, Gaizka Durana

**Affiliations:** 1 Communications Engineering Department, University of the Basque Country UPV/EHU, Ingeniero Torres Quevedo Plaza 1, E-48013 Bilbao, Spain; josu.amorebieta@ehu.eus (J.A.); gotzon.aldabaldetreku@ehu.eus (G.A.); joseba.zubia@ehu.eus (J.Z.); gaizka.durana@ehu.eus (G.D.); 2 Fundación Centro de Tecnologías Aeronáuticas (CTA), Bizkaia Technological Park, E-48170 Zamudio, Spain; josu.beloki@ctabef.com; 3 AOTECH, Advanced Optical Technologies S.L., E-48002 Bilbao, Spain; igarcia@aotech.es

**Keywords:** fibre bundle, reflective optical sensor, tip clearance, turbine, aero engine

## Abstract

Among the different available optical technologies, fibre bundle-based reflective optical sensors represent an interesting alternative for parameter monitorization in aero engines. Tip clearance is one of the parameters of great concern for engine designers and engineers. In the framework of this optical technology, three fibre-based reflective optical sensors have been compared. Two of them are custom designed and based on the same geometrical fibre arrangement, whereas the third one is commercially available and relies on a different geometrical arrangement of the fibres. Their performance has been compared in clearance measurements carried out during an experimental program followed at a transonic wind tunnel for aero turbines. The custom-designed solution that operates in the most sensitive part of its response curve proved to be by far the most reliable tool for clearance measurements. Its high resolution opens up the possibility to detect small blade features such as cracks, reflectivity changes, etc. that otherwise could not be tracked. These results show that the detection of unexpected features on blade tips may have an important effect on how the clearance is calculated, ultimately giving rise to corrective actions.

## 1. Introduction

Nowadays, the presence of turbines has notoriously increased in critical sectors such as military, civil transport, power plants, etc., which has derived from the interest of developing increasingly more efficient designs in order to improve both their efficiency, and durability.

The efficiency of turbines and their lifespan has been a field of study for years [1,2]. Those studies have resulted in a progressive improvement and refinement of turbines performance and design. The aforementioned improvement is related to different elements and/or parameters such as the tip clearance (TC), which is defined as the air gap between the most prominent part of any of the rotor blades and the inner part of the casing, also known as abradable [3]. Lowering TC allows extracting as much energy as possible from the incoming fluid. As the TC decreases, the sealing of the turbine stage improves, avoiding undesired air-leaks that reduce efficiency and performance. Overall, the benefits from TC reduction are lower fuel consumption [4,5], the decrease of contaminant emissions [6,7], and the increase of available payload [5], which are very interesting topics in the aeronautical industry, especially for civil aviation, which make the engines economically more profitable.

However, keeping the TC as low as possible may cause the blades to scratch the abradable. Even if the abradable is a material designed to withstand this situation, excessive friction may lead to a premature wear of certain engine parts and a reduction of the lifecycle of the engine. That effect would increase the running and maintenance costs [4]. In order to avoid this fact, active TC control systems have been developed [8]. 

Tip clearance measurement systems are required to constantly monitor the TC parameter to provide designers and engineers with reliable and precise information that could avoid potential issues or could be helpful for new turbine designs. Currently, several TC measurement systems are commercially available, which are based on different sensors: capacitive [9,10,11], eddy current [12,13,14,15,16], pneumatic [17], strain gauges [18], electromechanical [19], microwave [20,21,22,23,24], and optical sensors [25,26,27]. 

Each sensor type has different characteristics. On the one hand, both capacitive and eddy-current sensors have poor frequency response. On the other hand, the capacitive sensors are known for being low cost and robust, whereas the eddy-current sensors require targets with magnetic materials. Pneumatic sensors are insensitive to contamination, but they require considerable hardware. Strain gauges, along with their required wires, are usually installed in the blades, interacting with the air flow passing around and changing the mass balance of the turbine [28]. Electromechanical sensors have high resolution, but they only provide the measure of the closest blade of the rotor, and do not provide blade identification. Microwave sensors are not sensitive to contamination, but they need complex circuitry. Finally, optical sensors have fast response and small dimensions, and thus they are able to provide more detailed information, but they can be affected by dirt [29].

According to the conditions and limitations of measuring the TC in an aero turbine, where small sensor volume and high bandwidth are desirable, the use of an optical sensor is very appropriate. Among the available optical solutions for distance measurements, the most common techniques are based on Doppler effect, interferometry, triangulation, intensity modulation, and time of flight. 

The Doppler effect-based technique offers high positioning and temporal resolution over metallic and non-metallic blades [30]. Optical coherence tomography uses the interference back-reflected light from the blade tips and a frequency-shifted reference with variable time delay, making use of a low-coherence light source [31]. The triangulation technique emits a light beam towards the target with certain angle, and according to the target position, the reflected ray would hit the receiving sensor in a different position. However, this technique could experience problems with abrupt shapes [32,33]. Intensity modulation-based techniques rely on the dispersion of the laser beam energy. A laser beam is emitted, and as it travels, the light power per area decreases. So, the longer the beam travels the less power per area is sensed by a given photodetector. However, any increment in the power of the light source would be incorrectly interpreted as a target approach, even if the distance did not change. To avoid this, a source light with a stable power is needed. There is also a more complex variant, based on the Gaussian dispersion of the power of the traveling beam, where various fibre groups at different radial distances are needed to measure how the beam intensity is radially distributed [15,29,34,35,36,37,38]. This technology has more tolerance to the target angle compared to the intensity-based technique, but the sensor is more expensive and complex to build. The time of flight method relies on the time the light beam needs to travel from the sensor to the target and back. This technique is robust and reliable, but its accuracy is strongly dependent on the slope of the leading and trailing edge of the blade [17]. Therefore, after commenting about different techniques, the Gaussian dispersion-based technique is the best candidate for its light source power variation immunity and target angle variation tolerance.

The paper presents the performance comparison of three different optical sensors based on the Gaussian dispersion technique. First, the configuration of each of them is explained. Then, the experimental setup is described, as well as the test program carried out in a turbine rig at “Fundación Centro de Tecnologías Aeronáuticas” (CTA) facilities. Afterwards, the most relevant experimental data obtained from the three optical sensors are compared and discussed. Finally, the most relevant conclusions are summarized. 

## 2. Materials and Methods

### 2.1. Description of the Setup

**Figure 1:**
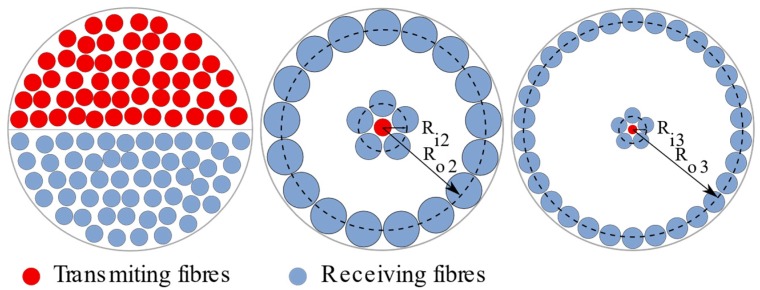
Cross-sections of fibre bundles from the three optical sensors under comparison: OS 1 (left), OS 2 (middle), and OS 3 (right). Images not to scale. R_i_ and R_o_ stand for inner and outer radius for OS 2 and OS 3 sensors.

The optical sensors (OS) under comparison all have optical fibre-based sensing heads. One is commercially available and the other two are based on custom-designed optical fibre bundles. The cross-section of each of them is shown in Figure 1. For the sake of clarity, from now on, from left to right they will be referred to as OS 1, OS 2, and OS 3. On the one hand, OS 1 (Philtec model RC171) consists of several transmitting and receiving fibres arranged in adjacent semi-circular pattern where the fibres are distributed randomly. Its specifications are available in Reference [39]. On the other hand, the two custom-designed sensors share the same geometrical structure, which is explained in References [3,36]. They are both based on two independents receiving fibre rings that are located at different radii surrounding the central transmitting fibre. They were designed based on the mathematical approach shown in References [37,38] but including the design constraints (maximum allowed diameter for the cross-section of the fibre bundle, expected tip clearance working range, etc.) imposed by the turbine specifications in order to have the most sensitive performance in their respective linear regions (Figure 4). With these considerations in mind, the manufactured custom designs that fulfilled the previously mentioned boundaries are shown in Figure 1. For both cases, OS 2 and OS 3, the transmitting fibre is a single-mode fibre with a numerical aperture (NA) of 0.12, and the inner ring of fibres (of radius R_i2_ = R_i3_ = 200 µm) is formed by five multimode fibres with an NA of 0.22. The OS 2 outer ring (of radius R_o2_ = 930 µm) is formed by 17 multimode fibres with a core diameter of 300 µm and an NA of 0.22, whereas the OS 3 (with a radius R_o3_ = 1800 µm) is formed by 30 multimode fibres with core diameters of 300 µm with an NA of 0.22. The main difference between both configurations lies in the number of the receiving fibres and ring radius on the outer ring: OS 3 has a bigger outer ring radius with a larger number of fibres on it than in the case of OS 2.

**Figure 2:**
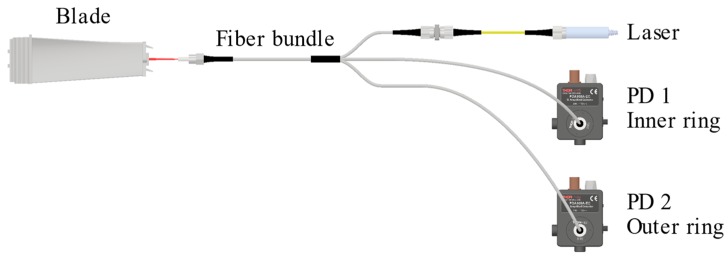
Schematic representation of the light-to-voltage conversion for OS 2 and OS 3. PD stands for photodetector. PD 1 and PD 2 are connected to inner and outer rings, respectively.

On the one hand, OS 1 integrates its own light source and a light-to-voltage converter by means of proprietary optoelectronics. On the other hand, OS 2 and OS 3 share a common continuous-wave (CW) laser light source (@660 nm wavelength) via a 50:50 optical fibre coupler. The light launches onto the passing blade by the central transmitting fibre, after being reflected on it, is gathered by each of the receiving fibre rings, the inner ring and outer ring, and converted into two voltage levels (V_1_ and V_2_) at photodetectors PD 1 and PD 2, respectively (please refer to Figure 2). The ratio of V_2_ to V_1_ is considered to minimize undesirable effects related to intensity fluctuations of the light source and reflectivity variations of the target surface [3]. Finally, all the voltage outputs are acquired simultaneously by a shared acquisition board. 

### 2.2. Assembly, Calibration, and Tests Program

**Figure 3:**
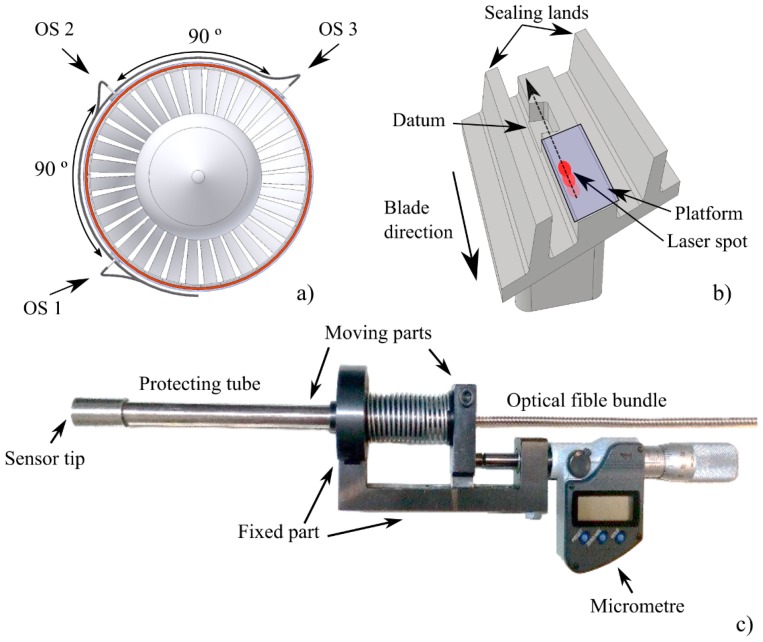
(**a**) Location of OS 1, OS 2, and OS 3 in the turbine casing. (**b**) Detail of a typical blade tip. (**c**) Side view of the tool used to locate precisely each sensor head with respect to the blade tips.

The sensors were installed in an aircraft turbine rig at the facilities of CTA in order to calibrate them specifically for the rig under test before the TC measurements were done. For that purpose, they were fixed into three of the four radially oriented inlets (see Figure 3a) that gave access to the platform located at the middle of the blade tip profile. The blade tip detail and the light path along it are shown in Figure 3b. The radial positioning was carried out with a precision positioner (Figure 3c). 

**Figure 4:**
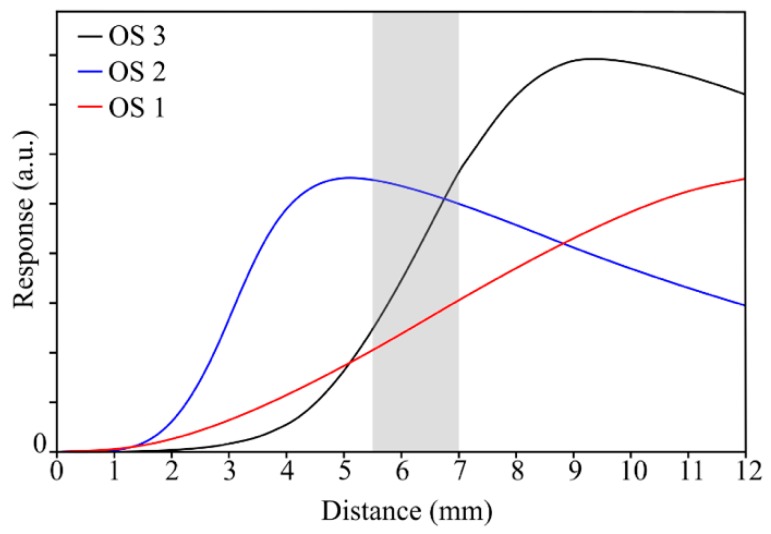
Calibration curve of each sensor. The shaded region shows the working region of interest.

The calibration curves of the optical sensors are shown in Figure 4. The shaded area represents the region of interest at which each sensor worked during the rig tests. The distance shown in Figure 4 represents the distance from the blade platform (Figure 3b) to the tip of the sensor installed in the engine casing. Notice that OS 1 and OS 3 are operating in their positive and more sensitive front slope region, whereas OS 2 does it in its less sensitive back slope region.

The tests were carried out in a wind tunnel commonly used for turbine testing [3]. Briefly, the test rig consisted of two stages: the first stage was formed by a 3.7 MW air compressor with a temperature control section, and the second stage was formed by a vacuum pump of 5 MW. In the pipe which connected both stages, an air flow rate up to 18 kg/s could be generated, a pressure up to 450 kPa, and a temperature up to 573 °C. The turbine was connected to a hydraulic brake to simulate changing loads.

The tests lasted from eight to ten continuous hours per day and they were repeated for two weeks in consecutive days. Every test point was recorded, since each one implied a change in the working conditions (pressure, rpm, etc.). All the sensors data were acquired by one acquisition board. Additionally, a once per revolution (OPR) signal for synchronisation and blade identification purposes was acquired. From the gathered data, the most representative results along with the corresponding discussion were analysed and the results along with a discussion are presented and explained in the following section.

## 3. Results and Discussion

**Figure 5:**
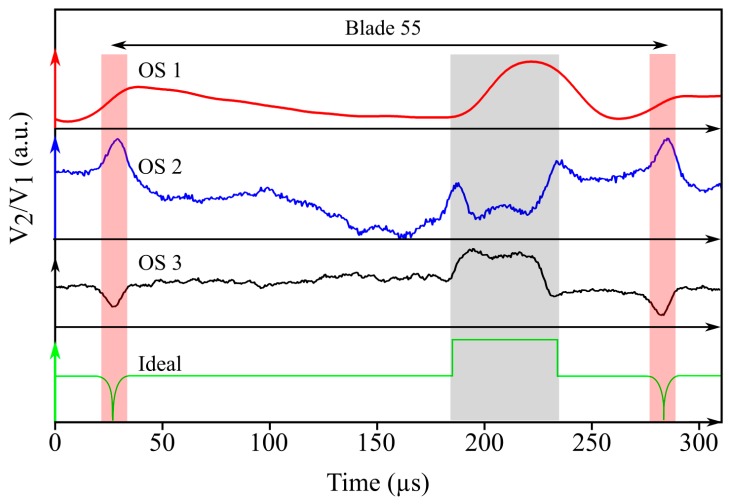
Comparison of sensors signals for a typical blade. The lower curve represents the ideal signal response. The datum and inter-blade spacing are highlighted with grey and red-shaded areas, respectively.

From the raw signals of the three sensors, different levels of blade information and detail may be obtained (see Figure 5). On the one hand, OS 1 shows a smoothed signal that, as we will see, is responsible for the slight delay observed in signal response to specific blade profile features. This fact makes it harder to detect and synchronise the different events found during a complete shaft turn. On the other hand, OS 2 and OS 3 are able to detect specific features, such as the datum and inter-blade spacing, more clearly and without delays, which results in an easier posterior data processing.

**Figure 6:**
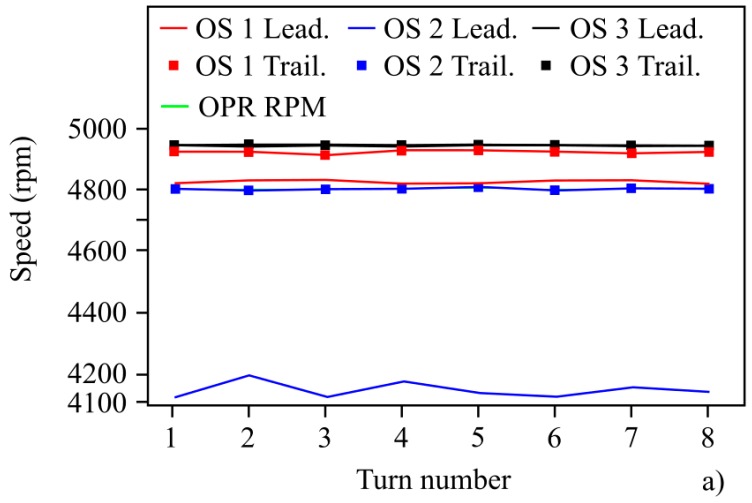

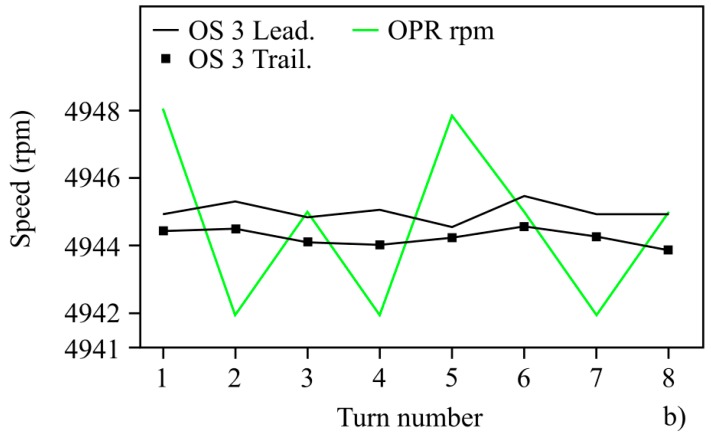
(**a**) Turbine instantaneous speed over several shaft turns measured by different optical sensors. The reference speed was defined by the OPR signal. (**b**) Comparison of the turbine instantaneous speed values given by OS 3 and the OPR signal.

The high resolution and sensitivity offered by OS 3 makes it possible to determine the passage of each blade precisely. The steep rising and falling edges in the waveform enable us to precisely determine the boundaries of the datum, and ultimately, their arrival times. More specifically, the derivative of the raw waveform yields a sequence of peaks associated with the leading and trailing boundaries of the datum and the inter-blade spacing that, after setting a convenient threshold value to the derivative, allows us to determine the passing times of the datum boundaries precisely. Therefore, within a complete turbine turn, it is possible to define as many instantaneous speeds as number of blades (94 in the turbine rig under test). For the discussion that follows, we define the instantaneous speed as the average speed (in rpm) over one turbine turn. Hence, the OPR signal directly gives it, whereas in the case of OS 1, OS 2, and OS 3, all blade-to-blade instantaneous speeds within one full turn must be averaged. If we consider the passing time of the trailing or leading edge of the datum as the reference value for defining the blade passing time, we can determine the instantaneous speed of the turbine. Figure 6a shows the turbine instantaneous speed determined according to the signals of each three optical sensors, using both the leading and trailing edges of the datum. It should be pointed out the inaccuracy of sensors OS 1 and OS 2 when trying to approach the reference rpm value given by the OPR signal. In fact, OS 1 and OS 2 using the datum leading edge to extract the blade-to-blade speed produced an output mean speed reading with an error of 0.4% and 2.9%, respectively, whereas using the datum trailing edge produced a speed error of 2.4% and 16%. In addition to that, the obtained result varies quite substantially depending on whether the leading or trailing edge has been considered for the determination of the arrival time. This is particularly remarkable in the case of OS 1, for which the leading edge converged to the expected value given by the OPR signal, whereas the trailing edge falls far apart from it. Regarding OS 3, a closer look at its performance revealed a very accurate behaviour irrespective of the chosen datum edge (see Figure 6b). Observe that all curves followed the same trend over eight revolutions of the rotor, but with the distinctive feature of the curve obtained from the OPR signal that has an edgy behaviour in contrast to the other two curves. The smoothed behaviour of the latter was a consequence of the averaging process of the 94 instantaneous speeds involved in a complete revolution, but gives a more realistic view of the actual speed behaviour of the turbine. This kept the mean error for the OS 3 below 0.009% and the maximum error around 0.07%.

**Figure 7:**
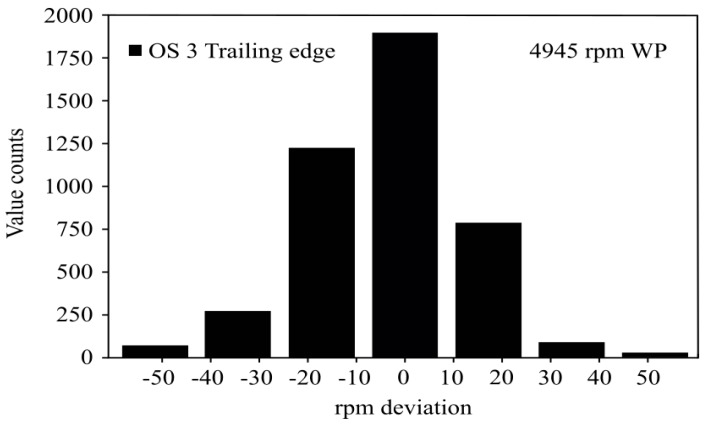
Histogram representing the deviation of the calculated instantaneous speed with respect to the nominal speed (4945 rpm) for the OS 3 sensor using the datum trailing edge. Data obtained from 52 consecutive shaft turns.

In order to appreciate further the accuracy provided by OS 3 against OS 1 and OS 2, a statistical analysis based on data from 52 complete turbine revolutions was carried out. More specifically, for each complete turbine turn, 94 blade-to-blade instantaneous speeds were determined and compared with the reference speed value set by the OPR signal. For the sake of graphical clarity, Figure 7 only shows the frequency distribution of the speed deviation corresponding to OS 3. The trailing edge of the datum was considered to define the passing time of each blade. Although not shown in Figure 7, data corresponding to OS 1 and OS 2 spread out over a wide range of values, whereas data from OS 3 were very concentrated around the nominal value. Additionally, outlier values also existed in the cases of OS 1 and OS 2.

A short summary of the statistical analysis is presented in Table 1. It highlights the lower performance of OS 1 and OS 2 compared to OS 3. The capability of the OS 3 to extract the blade-to-blade rotor speed, even along the duration of each turn, is outstanding. Similar results were obtained using the leading edge of the datum.

These observations are a direct consequence of the waveform quality of the acquired signals, among which OS 3 excels over the rest.

**Figure 8:**
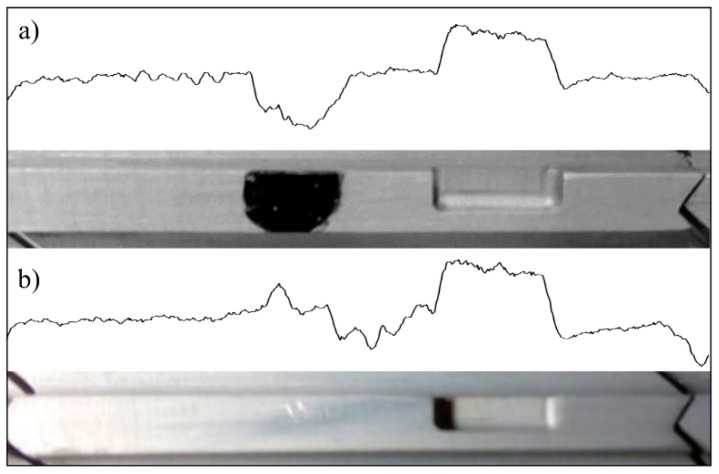
Waveform signals corresponding to OS 3 in two different cases: (**a**) a blade with a black-painted area on it. (**b**) A blade with variable reflectivity along it.

The high resolution and sensitivity of OS 3 also provides the ability to identify unexpected features in the blades through the corresponding waveform analysis. Two representative examples of this ability are shown in Figure 8. 

**Figure 9:**
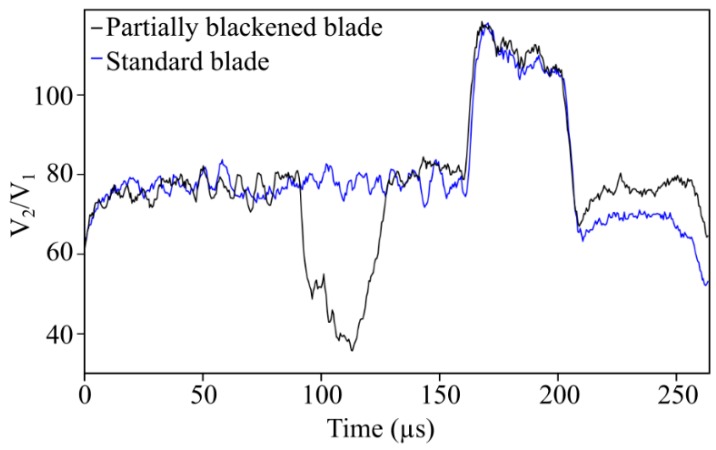
Comparison between a standard blade (blue curve) and a blade with an unexpected feature on it (black curve). In this case, the feature corresponds to a small blackened area on the long platform of the blade.

The first example (see Figure 8a) shows the waveform signal corresponding to a blade that has a black marker dot on it. The waveform shows a clear drop in light intensity as a consequence of the lower reflectivity of that blackened area. Something similar occurs in the second example (see Figure 8b) where the surface reflectivity varies along the long flat platform and the waveform responds accordingly. It is worthy of note that the waveform response to the passage of standard blades (blades without any unexpected features) is quite constant in the long and short platform areas of the blades. The comparison between the waveform response to a standard blade and to a partially blackened blade is shown in Figure 9. At this point, it is important to bring to the reader’s attention that the signal comparative has not been extended to OS 1 and OS 2 as they struggle to distinguish between a standard blade and a blade with a certain feature on its tip. Coming back to Figure 9, it should also be pointed out that the signal response to the passage of a typical blade, which is quite constant over the long and short platforms of the blade, has a low-amplitude, high-frequency fluctuation superposed on it. Although at first sight it may seem a random noise contribution to the actual waveform signal, it actually represents the response to a surface with a certain roughness level on it. 

**Figure 10:**
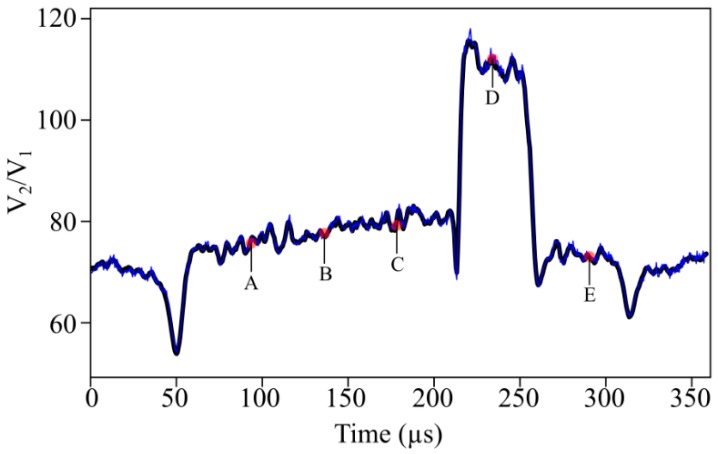
Signal response of OS 3 to the passage of a typical blade over 1100 revolutions. The black curve represents the average response curve, whereas the blue curve corresponds to a single revolution.

As evidence of the previous statement, Figure 10 shows the response of OS 3 to the passage of a typical blade over 1100 turns of the turbine. The almost perfect overlapping of the successive signals suggests that the low-amplitude, high-frequency fluctuations were mainly related to surface features and irregularities of the blade tip, which again confirm the low noise and high stability of the sensor. As a quantitative measure of it, Table 2 shows the average and standard deviation values of the signal at different points distributed in an equidistant way along the length of a typical blade (points A, B, C, D, and E in Figure 10). Observe that the standard deviation did not exceed—in any case—5% of the corresponding average value. 

In the tests carried out at the CTA facilities, the TC was the parameter of interest to be monitored. The TC is defined as the air gap existing between the abradable coating and the most prominent part of each blade. Its determination requires a specific processing of the signal. For the analysis that follows, we will concentrate on OS 3 as its performance stands out over the other two optical sensors. The aforementioned signal treatment (for a standard blade) consists in averaging the data from a predefined section of it. More specifically, the average is carried out over the set of data corresponding to the long platform of each blade where, as commented previously, the signal level remains quite constant. However, in the case of the few non-standard blades, i.e., blades with unexpected features, such as shown in Figure 8, the TC value gets miscalculated due to the effect of those features on the signal shape and level. In those cases, some corrective actions may be taken to compensate for the harmful effects on the TC value. In order to make it clearer, let us consider blade number 54. The numbering refers to the number assigned to each blade according to its arrival position starting to count from the OPR signal. Therefore, blade 54 is the 54th blade to be detected by OS 3. In the case of that blade, if we do not consider any corrective action, its TC value results to be the minimum blade TC among all blades, i.e., the turbine TC. However, if we consider a corrective action based on excluding those data points coming from the problematic region of the blade, so that we end up with a smaller dataset for the averaging process, the new TC value increases, and the blade ceases to be the critical one (see Table 3). 

**Figure 11:**
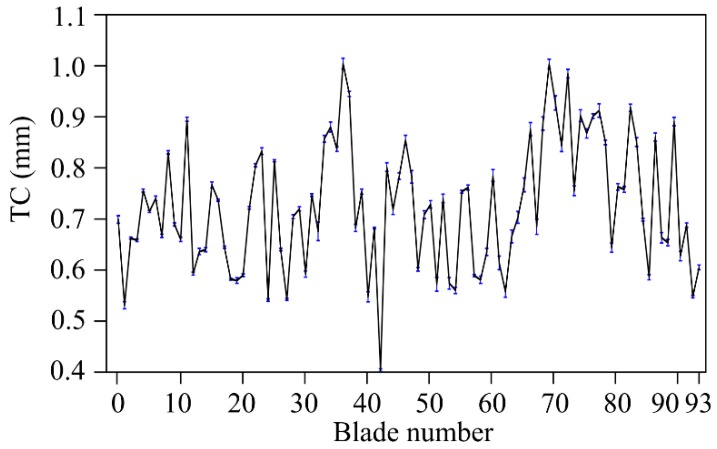
Tip clearance values of individual blades at 3627 rpm with error bars that account for the standard deviation over 20,000 revolutions.

As a proof of the stability and low noise brought by OS 3, Figure 11 shows the TC map measured with OS 3 corresponding to a certain engine working point (WP) at 3627 rpm. The variability of the TC values over time (20,000 engine revolutions), shown as vertical error bars, give rise to a worst case of 20 µm within the same WP, and the average value over the 94 blade is below 7 µm. 

**Figure 12:**
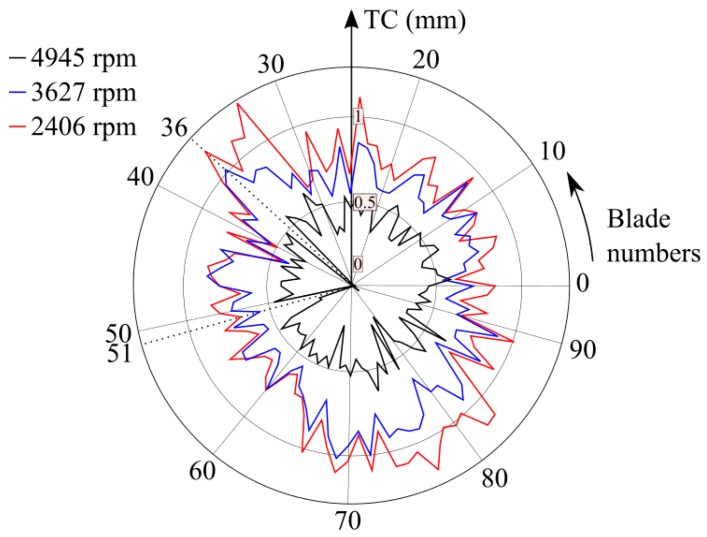
TC values of each of the 94 blades obtained with OS 3 at three different WPs.

The TC mapping of all blades corresponding to three WPs of the turbine in ascending order of rpm is shown in Figure 12. On those non-standard blades, for the correct determination of the corresponding TC, the aforementioned corrective action was applied. Results indicated that the higher the rotational speed was, the lower the TC. This was due to the centrifugal forces that make the blades stretch, and therefore, approach the casing. 

## 4. Conclusions

We have reported on a performance comparison between three intensity-modulated optical fibre-based sensors (one commercial with semi-circular fibre distribution, OS 1, and two custom-designed with concentrical ring distribution, OS 2 and OS 3) for aero engine applications. More specifically, the sensors have been tested in the wind tunnel available at the CTA facilities for TC measuring during turbine testing.

The OS 2 and OS 3 custom-designed sensors both had a central transmitting single-mode fibre and an inner ring formed by five multimode receiving fibres (core diameters of 200 µm and NAs of 0.22). On the other hand, their outer rings were built using larger multimode fibres (core diameters of 300 µm and NAs of 0.22), in each case with different amounts of multimode fibres in order to meet different working distance specifications. Each sensor had different calibration curves with two linear regions (front slope and back slope). The sensors worked in different regions depending on the distance to the target. The custom-designed sensor OS 3 was designed with the help of a custom simulation program developed within the research group. That design was developed in such a way that the sensor would be working in the front slope region of its response curve for the expected target distances on this test. During the test, OS 3 offered the best performance due to the higher stability, lower noise level, higher resolution and sensitivity, and great repeatability, which allowed for more accurate measurements. The TC values over 20,000 engine revolutions were registered showing a mean variability below 7 µm for each individual blade turn after turn. The signal provided by the OS 3 can be straightforwardly related to the physical shape of the blade, detecting the different features as the interlock and the datum. That feature makes it possible an easier and more reliable development of different software-based solutions, such as the blade-by-blade speed detection system that has been proven here, whose results provided a smoother rotor speed behaviour. That makes the OS 3 sensor stand out as the most interesting sensor of the test. 

Both custom-designed sensors, OS 3 and OS 2 (even though the signal in the latter is noisier), can detect small surface imperfections as scratches or dirt that are not recognized by the OS 1 sensor. This capability enables detecting blades that cause anomalous TC output value readings. In such situations, the software can be adapted to overcome that problem. Thus, the probed ability to detect surface irregularities in certain blades makes it also useful for health monitoring analysis. 

Our optical bundle is a great sensor for high speed TC measurement systems. It can also be customized for different applications where a small distance has to be measured. Therefore, we believe that this sensor may be appealing in high-precision applications, where non-contact measurement, small dimensions, and electromagnetic immunity are required, such as aeronautical engines, gas and oil facilities, etc.

## Figures and Tables

**Table 1:** Statistical analysis of OS 1, OS 2, and OS 3 working at 4945 rpm using the trailing edge of the datum feature.

Sensor	Mean (rpm)	Standard Deviation of the Mean (rpm)
OS 1	−21.37	±3.06
OS 2	−141.74	±7.13
OS 3	0.09	±0.40

**Table 2:** Signal stability over 1100 turbine turns at five points distributed equally along the length of a typical blade.

Point	Mean Value and Standard Deviation (V_2_/V_1_)	Relative Standard Deviation (%)
A	74.21 ± 0.53	3.5
B	77.91 ± 0.54	2.8
C	79.28 ± 0.64	3.3
D	114.62 ± 0.73	1.3
E	73.05 ± 0.64	4.6

**Table 3:** Tip clearance values at different rpms for a non-standard blade (blade 54), without and with the correction factor.

Rpm	TC without Correction (mm)	TC with Correction (mm)
2406	0.2	0.6
3627	0.3	0.5
4945	−0.2	0.4
